# B Cell Dysregulation in Common Variable Immunodeficiency Interstitial Lung Disease

**DOI:** 10.3389/fimmu.2020.622114

**Published:** 2021-02-05

**Authors:** Erik M. Matson, Miranda L. Abyazi, Kayla A. Bell, Kevin M. Hayes, Paul J. Maglione

**Affiliations:** Pulmonary Center, Section of Pulmonary, Allergy, Sleep & Critical Care Medicine, Department of Medicine, Boston University School of Medicine, Boston Medical Center, Boston, MA, United States

**Keywords:** common variable immune deficiency, CVID, GLILD, interstitial lung disease, TACI, BAFF-R, rituximab, B cell activating factor

## Abstract

Common variable immunodeficiency (CVID) is the most frequently diagnosed primary antibody deficiency. About half of CVID patients develop chronic non-infectious complications thought to be due to intrinsic immune dysregulation, including autoimmunity, gastrointestinal disease, and interstitial lung disease (ILD). Multiple studies have found ILD to be a significant cause of morbidity and mortality in CVID. Yet, the precise mechanisms underlying this complication in CVID are poorly understood. CVID ILD is marked by profound pulmonary infiltration of both T and B cells as well as granulomatous inflammation in many cases. B cell depletive therapy, whether done as a monotherapy or in combination with another immunosuppressive agent, has become a standard of therapy for CVID ILD. However, CVID is a heterogeneous disorder, as is its lung pathology, and the precise patients that would benefit from B cell depletive therapy, when it should administered, and how long it should be repeated all remain gaps in our knowledge. Moreover, some have ILD recurrence after B cell depletive therapy and the relative importance of B cell biology remains incompletely defined. Developmental and functional abnormalities of B cell compartments observed in CVID ILD and related conditions suggest that imbalance of B cell signaling networks may promote lung disease. Included within these potential mechanisms of disease is B cell activating factor (BAFF), a cytokine that is upregulated by the interferon gamma (IFN-γ):STAT1 signaling axis to potently influence B cell activation and survival. B cell responses to BAFF are shaped by the divergent effects and expression patterns of its three receptors: BAFF receptor (BAFF-R), transmembrane activator and CAML interactor (TACI), and B cell maturation antigen (BCMA). Moreover, soluble forms of BAFF-R, TACI, and BCMA exist and may further influence the pathogenesis of ILD. Continued efforts to understand how dysregulated B cell biology promotes ILD development and progression will help close the gap in our understanding of how to best diagnose, define, and manage ILD in CVID.

## Introduction

Primary antibody deficiencies (PADs) are the most prevalent form of immunodeficiency and are defined by disruption of a patient’s ability to generate functional antibodies. They are further classified by the mechanism of disruption and type of antibody affected. For example, X-linked agammaglobulinemia is an antibody deficiency defined by a reduction in all antibody classes due to a severe block in B cell differentiation, and hyper IgM syndrome is a deficiency characterized by defective B cell isotype class switching that results in lower levels of IgG and IgA, and higher IgM (1–3). The lack of a complete antibody arsenal typically predisposes PAD patients to recurrent bacterial and viral infections; however, the severity and prevalence of symptoms varies with type of PAD as well as individual manifestations of those with the same PAD.

The most prevalent symptomatic PAD is common variable immune deficiency (CVID) which is classified by profound reduction in IgG as well as IgA or IgM due to impaired B cell differentiation (4). Affecting 1:25,000 individuals, patients are typically diagnosed between the ages of 20 and 40 (5). Immunoglobulin replacement therapies can be used to limit infections, however about half of CVID patients develop non-infectious complications such as autoimmunity, lung and/or gastrointestinal disease, and malignancy despite this therapy (6). Moreover, these non-infectious complications occur in CVID more frequently than other forms of PAD for reasons that are poorly understood (7, 8). This suggests the presence of genetic, immunological, and/or environmental factors, and not simply antibody deficiency alone, drive the development of inflammatory complications in PAD. Yet, these complex etiologies remain poorly understood. Consequently, non-infectious complications are the leading cause of morbidity and mortality in CVID (9, 10).

The lung, as a mucosal surface regularly exposed to exogenous pathogens, is one of the organs most affected by the infectious and non-infectious complications of CVID. Upper respiratory tract infections by encapsulated bacteria are common in patients, leading to airway inflammation, impaired host defense, permanent tissue damage, and frequently bronchiectasis - an irreversible dilation of the bronchial airways (11). While bronchiectasis is likely the most common pulmonary complication of CVID, interstitial lung disease (ILD) also occurs in about 1 out of 3 CVID patients and accounts for a larger percentage of mortality (9, 10, 12). Radiological findings that distinguish CVID ILD typically include pulmonary nodules, ground glass opacities, and mediastinal lymphadenopathy (13). Additionally, biopsies typically reveal benign lymphoproliferation and granulomatous inflammation leading this form of interstitial lung disease to be labeled granulomatous-lymphocytic interstitial lung disease (GLILD) (1, 13). The exact cause of ILD in CVID remains unclear and does not require the presence of bronchiectasis or history of pneumonia, suggesting that infection is not an underlying cause in many cases (14). Immunoglobulin replacement therapy typically does not ameliorate the development of ILD in CVID, and current therapeutic approaches rely on immunomodulatory drugs (15). While treating ILD, these immunomodulatory drugs may also increase the risk of infection or malignancy in these patients already vulnerable for these complications, particularly because a therapeutic endpoint is often unclear (16). Greater understanding of ILD pathogenesis in CVID is needed to develop safer and more effective therapeutic approaches.

Perhaps a key to understanding ILD pathogenesis in CVID is the fact that it frequently occurs together with other non-infectious complications, like autoimmune cytopenia and splenomegaly, which are driven by mechanisms of immune dysregulation (17). Additionally, there are a number of monogenic antibody deficiency syndromes that present with ILD of a similar pathology to that seen in CVID patients (18). These include patients with gain-of-function mutations of *PI3KD* that develop the CVID-like activated PI3Kδ syndrome defined by lymphoid hyperplasia, which can affect the airways. Activated PI3Kδ syndrome can be ameliorated by rapamycin, which reduces resultant hyperactive mTOR signaling in lymphocytes, or targeted inhibition with the PI3Kδ inhibitor leniolisib (19, 20). Similarly, patients with genetic deficiency of cytotoxic T-lymphocyte-associated protein 4 (CTLA-4) or a protein vital for its vesicular trafficking, lipopolysaccharide (LPS)-responsive and beige-like anchor protein (LRBA), develop inflammatory complications that are responsive to CTLA-4-Ig, known as abatacept (21, 22). These examples highlight the potential of precision immunomodulatory treatments for ILD as well as other non-infectious complications of CVID based upon identification of an underlying genetic lesion.

Despite CVID being defined by impaired antibody production, B cells appear to play an important role in ILD pathogenesis. Pulmonary B cell hyperplasia is a defining feature of CVID ILD, particularly in patients with biopsy proven follicular bronchiolitis, lymphocytic interstitial pneumonia, and nodular lymphoid hyperplasia of the lungs (23). Notably, ILD occurs far less commonly in X-linked agammaglobulinemia, a form of PAD where B cells are absent (7). Numerous studies have found B cell-depletive therapy with rituximab to be efficacious for CVID ILD (23–27). We conducted the largest study of rituximab monotherapy for CVID ILD, finding clear efficacy of this intervention over supportive care (28). ILD recurred after rituximab in about 1/3^rd^ of subjects, but this recurrence could be limited by additional immunosuppression with azathioprine or mycophenolate. ILD recurrence was associated with increased levels of B cell activating factor (BAFF) in the blood and lungs, a key cytokine for B cell activation and survival (28). While these results do not prove that B cells are pathogenic in CVID ILD, they provide justification for deeper consideration and further research efforts to understand how these lymphocytes may contribute to disease. In the effort to summarize our understanding of how B cells may contribute to CVID ILD, we will review mechanisms of B cell dysfunction described in CVID and non-CVID lung diseases alike. We apply particular focus upon BAFF-related B cell biology given the considerable research in CVID and other lung diseases that has been recently conducted.

It is important to note that not all ILD found in CVID may be the same. It has been suggested that there are diverse forms of ILD afflicting CVID patients (29). We have found evidence of B cell hyperplasia and heightened BAFF responses in CVID, specifically with biopsy-proven forms of benign lymphoproliferative interstitial lung disease. This is a spectrum of pulmonary pathology that starts with follicular bronchiolitis, when disease is limited to peribronchial areas, and progresses to lymphocytic interstitial pneumonia and nodular lymphoid hyperplasia, when inflammation becomes more diffuse within the lung parenchyma (30). CVID ILD can also manifest as other types of pathology, such as non-specific interstitial pneumonia, prominent granulomatous inflammation, or organizing pneumonia (12, 14). It may be important to confirm ILD by performing lymphocyte phenotyping of biopsies to gain a specific pathology diagnosis, like lymphocytic interstitial pneumonia, rather than label all forms of presumed ILD on CT scan as GLILD and treat them the same. It is likely that CVID ILD pathology with prominent B cell follicles, such as follicular bronchiolitis and lymphocytic interstitial pneumonia, may be more responsive to B cell-targeted therapy. Variability among CVID ILD pathology may mean that some cases are more responsive to BAFF or B cell-targeted therapy than others.

## Biology of BAFF and Its Receptors

BAFF and a proliferation-inducing ligand (APRIL), are members of the tumor necrosis factor family of ligands that share receptors to promote activation and survival of B cells. BAFF and APRIL are elevated in the blood of CVID patients (31, 32). BAFF may contribute to lung disease in CVID as its levels were found to be highest in CVID patients with progressive ILD (28). APRIL levels were not found to be also elevated in this study. A variety of cell types are capable of producing BAFF in response to type I and type II interferons as well as pattern recognition receptor engagement, including dendritic cells, monocytes, and neutrophils (33). BAFF is expressed as a type II transmembrane protein that is processed at a furin cleavage site to release soluble BAFF (33, 34). Upon release from the cell membrane, BAFF can assemble into homotrimers or oligomeric, capsid-like 60-mers (35). Alternative splicing of BAFF generates a shorter isoform (ΔBAFF) that is co-expressed and associates with BAFF but interferes with proteolytic cleavage at the membrane (36). Thus, soluble BAFF can have distinct functional impact upon B cells depending on its abundance, multimeric state, and isoform.

The effects of BAFF are influenced by the specific receptor it binds. BAFF can signal *via* three receptors, BAFF receptor (BAFF-R), transmembrane activator and CAML interactor (TACI), and B cell maturation antigen (BCMA), while APRIL signals through TACI and BCMA only (**Table 1**) (37). BAFF receptors are differentially expressed across developmental subsets of B cells to regulate intracellular signaling pathways related to B cell activation, survival, and maturation (37–39). Expression of BAFF-R is absent on pre-B cells in the bone marrow until development into immature B cells, coinciding with establishment of BAFF-R as the predominant BAFF receptor in naive and transitional B cells (39). TACI expression increases with development into marginal zone and memory B cells as well antibody producing cells (38, 40). Expression of BCMA is mainly restricted to plasma cells (38, 41–43).

**Table 1:** Important characteristics of the receptors for BAFF.

	BAFF-R (*TNFRSF13C*)	TACI (*TNFRSF13B*)	BCMA (*TNFRSF17)*
**B cell subset expression**	Naïve & transitional B cells	Marginal zone & class-switched memory B cells	Plasma cells
**Ligands**	BAFF trimer, BAFF 60mer	BAFF 60mer, APRIL, HSPGs	BAFF, APRIL
**TRAF Interactions**	TRAF3 TRAF6 TRAF2 (thru TRAF3)	TRAF2 TRAF3 TRAF5 TRAF6	TRAF1 TRAF2 TRAF3 TRAF5 TRAF6
**Signaling pathways**	Non-canonical NF-κB Canonical NF-κB PI3K-Akt	Canonical NF-κB NFAT MyD88-dependent CSR	Canonical NF-κB
**Effects upon B cells**	Pro-survival Enhanced proliferation Resistance to apoptosis	Cell cycle arrest Apoptosis TI class switching to IgG, IgA Plasma cell differentiation	Survival of plasma cells
**Extracellular CRDs**	1 (shorter)	2	1
**Soluble receptor processing**	ADAM10, ADAM17 (BAFF & TACI-dependent)	ADAM10, γ-secretase, ADAM17	γ-secretase

**Figure 1:**
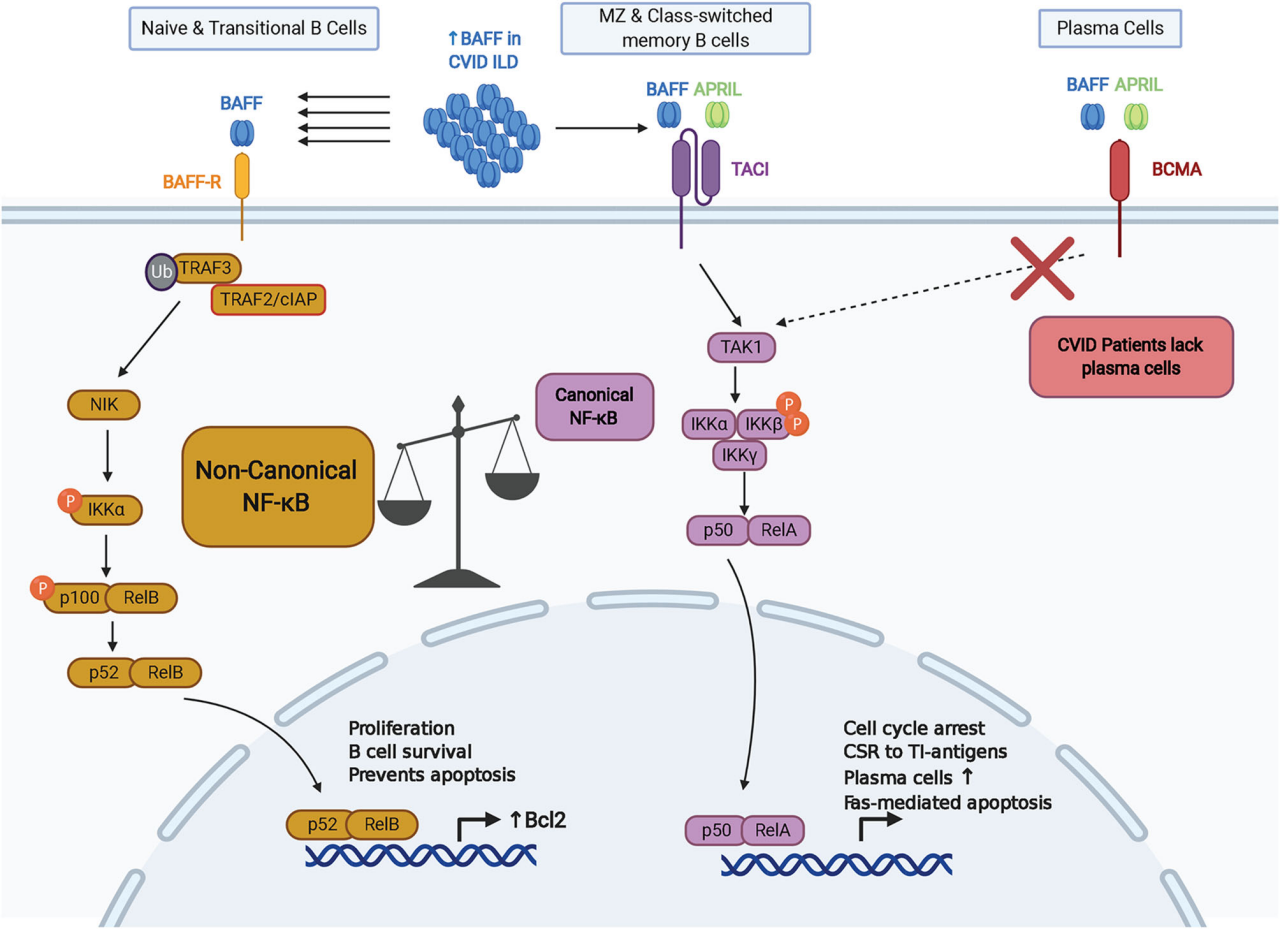
Key aspects of BAFF-R, TACI, and BCMA signaling within the context of CVID. BAFF-R is distinguished by its ability to signal *via* the non-canonical NF-kB pathway to induce Bcl-2 and other pro-survival factors. Lack of memory B cells and plasma cells expressing TACI and BCMA in CVID may increase signaling *via* BAFF-R. CSR, class-switch recombination; TI, T-independent.

Along with differences in expression during B cell maturation, there are distinguishing features regarding BAFF-R signaling compared to other receptors for BAFF (**Figure 1**). In addition to activating the canonical NF-κB and phosphoinositide 3-kinase pathways, BAFF-R engagement of trimeric or oligomeric BAFF activates the non-canonical NF-κB pathway and upregulates expression of proteins in the Bcl-2 family that enhance B cell survival (44–46). Non-canonical NF-κB signaling requires activation of NF-κB-inducing kinase (NIK), a kinase that is targeted for constitutive degradation while in complex with TNF receptor-associated factor 3 (TRAF3) in unstimulated B cells (47, 48). Ligation of BAFF to BAFF-R induces the targeted degradation of TRAF3, allowing NIK to accumulate and induce IκB kinase (IKKα)-dependent cleavage of p100 into p52 which associates with RelB to alter transcriptional activity (49–54). TRAF molecules such as TRAF2, TRAF3, TRAF5, and TRAF6, are recruited to the intracellular domain of BAFF receptors to mediate downstream signaling pathways in B cells through the canonical and non-canonical NF-κB pathways, AP-1 signaling, and MyD88-dependent class switch recombination in cooperation with TLRs (51, 55–58). B cell survival is also enhanced through the cooperation of BAFF-R with CD19 in regulating the activity of phosphoinositide 3-kinase (59). BAFF-R has greater affinity for BAFF compared to TACI and BCMA (60, 61). Due to its ability to promote survival through the non-canonical NF-κB and phosphoinositide 3-kinase pathways, expression from early stages of B cell maturation, and high affinity for BAFF, BAFF-R is positioned as a chief mediator of BAFF activity. Further efforts are needed to determine how significant the role of BAFF-R is in the pathogenesis of CVID-related complications.

Unlike BAFF-R, TACI signal activation requires binding to a higher-order oligomeric BAFF complex, such as the BAFF 60mer (62). BAFF signaling through TACI activates the canonical NF-κB pathway and upregulates expression of genes involved with cell cycle arrest, cell death, and class switch recombination (CSR) in response to T cell-independent (TI) antigens (45, 63, 64). In line with the role of TACI in TI responses, BAFF and APRIL induce IgG and IgA CSR *via* TACI through MyD88 (64). TACI interacts with mechanistic target of rapamycin (mTOR) *via* MyD88 to contribute to TACI-mediated NF-κB activation, association with TLRs, and IgG class switching in response to TI antigens (65). TACI also appears to have a regulatory role in antibody production from B cells stimulated with BAFF and CD40, which indicates a homeostatic role in regulating T cell-independent versus T cell-dependent antibody production (66). TACI can also signal through the nuclear factor of activated T cells (NFAT) pathway (67). Alternative splicing of TACI transcripts can generate a short isoform that induces strong activation of the NF-κB pathway and has distinct localization within B cells compared to the full-length isoform (68, 69). Importantly, TACI signaling promotes expression of BLIMP-1, a transcription factor that induces cell cycle arrest and plasma cell differentiation by inhibiting expression of Bcl-6 and Pax5 (63, 70). Interestingly, Pax5 has been characterized as a lineage biomarker for a subset of rituximab-treated B cell lymphoma patients who relapse with CD20-negative B cells (71–74). However, the role of Pax5 in the development and progression of non-infectious complications in CVID remains to be characterized.

## BAFF and Its Receptors in CVID

**Figure 2:**
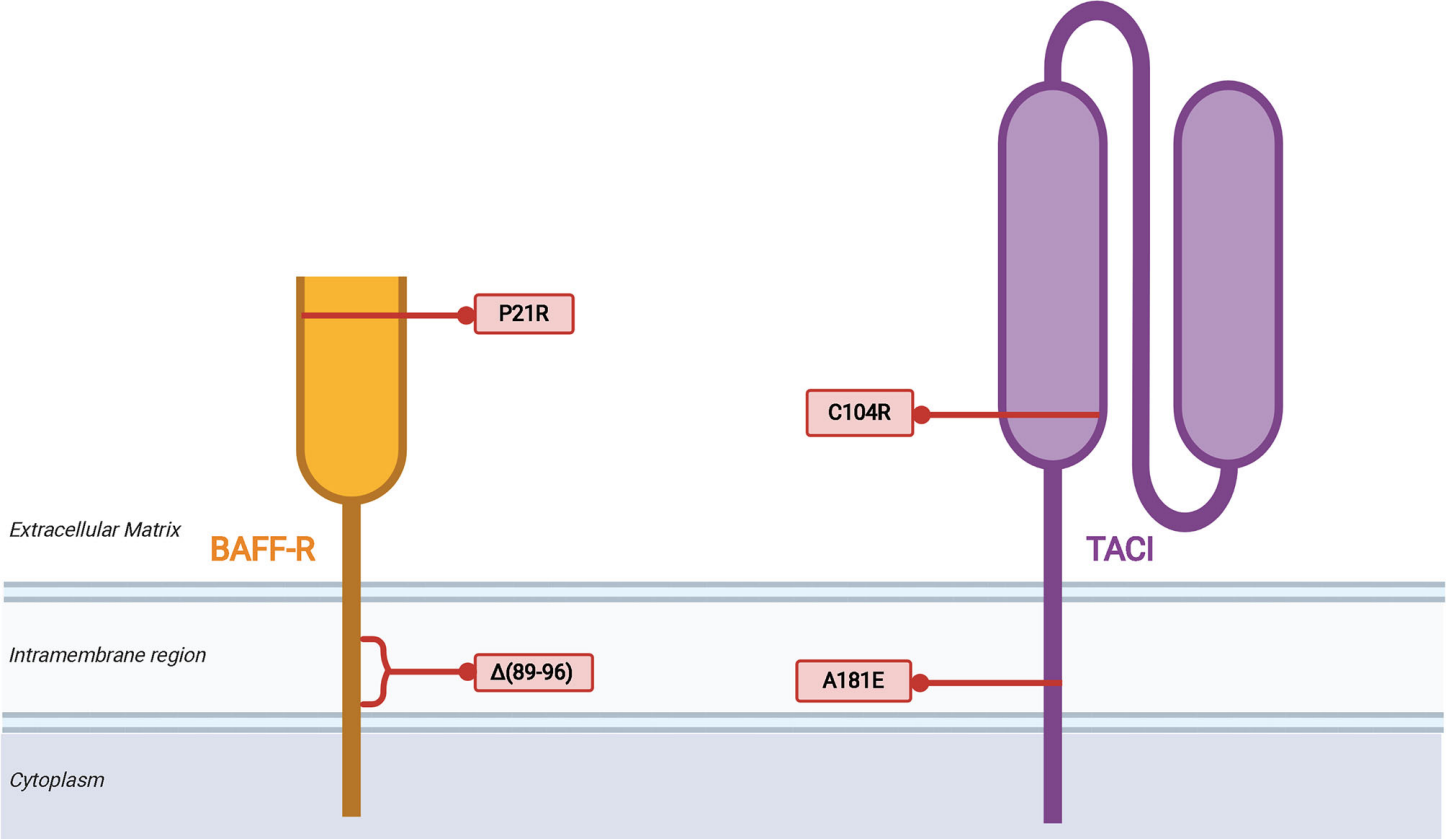
Mutations of *TNFRSF13B* (TACI) associated with CVID. The variants listed are limited to the two most common, C104R and A181E, which are discussed in the text, as well as two other illustrative examples of how disruption of TACI can impair B cell function. Proposed mechanisms of biochemical disruption of certain variants included.

Germline mutations in *TNFRSF13B*, the gene that encodes TACI, are observed in 5-10% of CVID patients (75, 76). TACI-deficient patients are known to have an increased rate of autoimmunity and lymphoproliferative disease in CVID in association with increased autoreactive B cell selection and survival (77, 78). There may a greater risk of progressive ILD in CVID patients with certain TACI mutations compared to other CVID patients (28). The C104R and A181E variants are the most common variants in TACI that are considered likely pathogenic (**Figure 2**). The C104R mutation disrupts a disulfide bond in the extracellular cysteine rich domain 2 (CRD2) to diminish TACI ligand binding capacity and TACI-mediated activation of canonical NF-κB signaling (79). The A181E TACI variant affects the CAML binding site located in the transmembrane domain does not interfere with ligand binding or surface expression but fails to activate NF-κB signaling (79). Several other CVID-associated genetic variants of TACI have been identified in clinical settings and further characterization of these variants may provide insight into TACI’s role in regulation of the BAFF/APRIL signaling axis in CVID and other diseases (75–77, 79–82). A global cohort analysis revealed that although mutations in *TNFRSF13B* are prevalent in CVID and healthy populations, there is an excess of rare derived alleles of *TNFRSF13B* in CVID cohorts compared to healthy individuals of the same population, indicating that defects in TACI are contributory toward manifestations of CVID (80). However, given the prevalence of the same variants in healthy populations, *TNFRSF13B* mutations are likely disease-modifying rather than disease-causing.

Regarding BAFF-R, a homozygous in-frame deletion that results in the loss of eight amino acids within the transmembrane region was identified in siblings with hypogammaglobulinemia (83). The two siblings had reduced serum IgG and IgM, but normal level of IgA. Class-switched memory B cells were lacking in these patients, and they did not have a medical history of autoimmune or lymphoproliferative complications. Also, a P21R variant of BAFF-R has been identified that interferes with BAFF-R complex formation, has reduced capacity to bind BAFF, and impairs BAFF-mediated NF-κB2 activation (84). B cells from patients with the BAFF-R P21R mutation lacked an increase in cell number and IgM secretion in response after stimulation with CpG DNA, anti-IgM, and BAFF. The BAFF-R P21R allele is found in 10.2% of CVID patients and 6.7% of healthy controls. Three additional heterozygous BAFF-R variants have been identified in a CVID cohort, all of which are present in healthy controls as well and their role in CVID remains to be defined (85).

## Soluble BAFF Receptors

**Figure 3:**
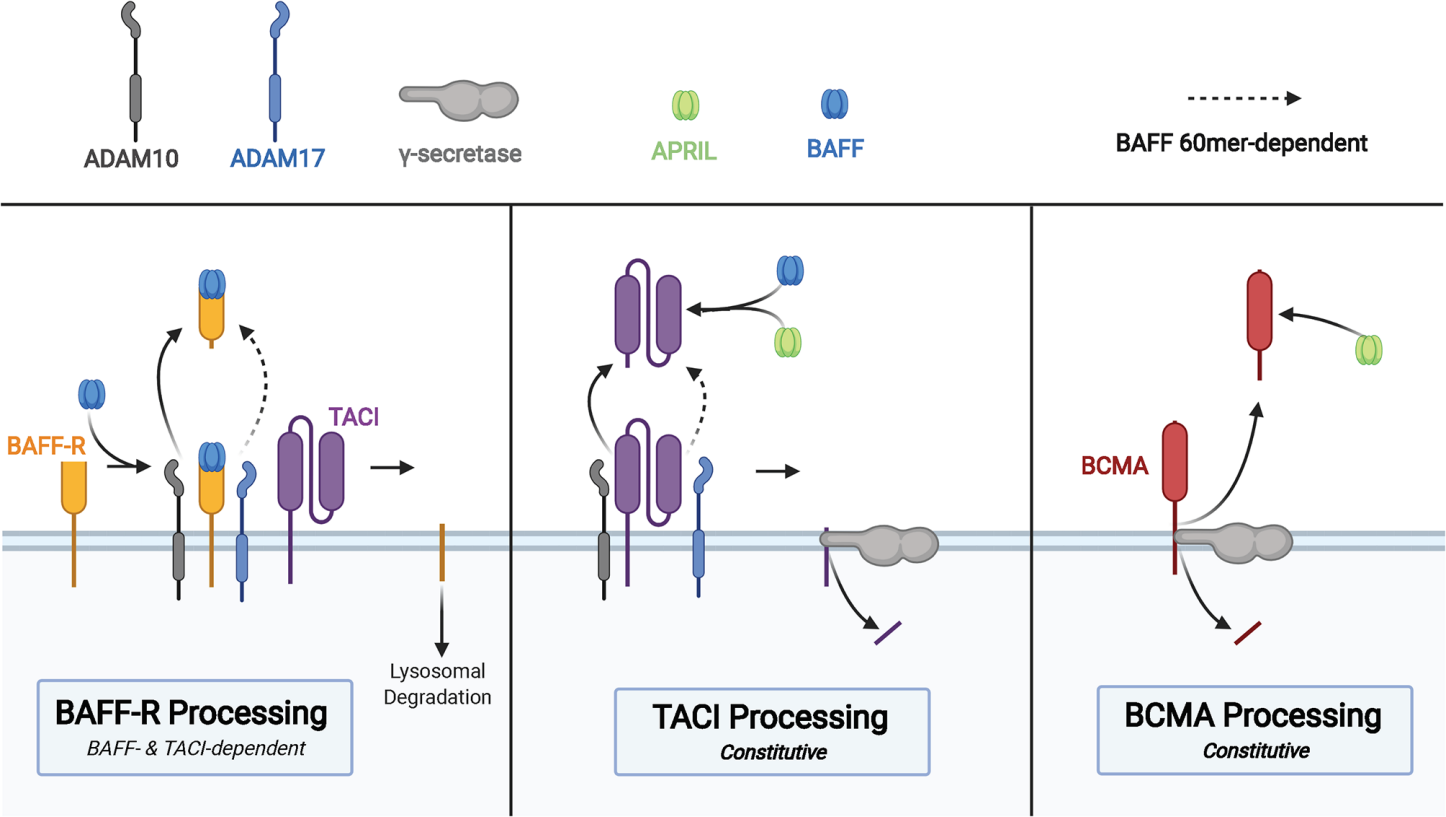
Membrane processing of human BAFF receptors. Cleavage of the BAFF-R ectodomain is induced by BAFF binding in cells that co-express TACI. Processing of BAFF-R by ADAM10 is induced by binding to BAFF trimers and binding of BAFF 60mer to BAFF-R induces ADAM17 processing of BAFF-R. The membrane-bound C-terminal fragment of BAFF-R is degraded in lysosomes after cleavage of the ectodomain. TACI is cleaved in a constitutive manner by ADAM10, followed by cleavage of the membrane-bound C-terminal fragment by γ-secretase. sTACI exhibits homotypic assembly and binds to BAFF and APRIL to reduce NF-κB activation and B cell survival, with TACI-Fc demonstrating similar capabilities. BCMA is constitutively cleaved by γ-secretase to release sBCMA consisting of the ectodomain and a portion of the transmembrane domain of BCMA. sBCMA is a decoy for APRIL-induced NF-κB activation but does not block BAFF-mediated NF-κB activation. However, BCMA-Fc is capable of binding both APRIL and BAFF to block NF-κB activation.

Each of the three BAFF receptors can be proteolytically processed to generate soluble molecules that function as decoy receptors in circulation (**Figure 3**). These soluble BAFF receptors add another layer to regulation of BAFF and APRIL-mediated homeostasis in B cells, prompting investigations into their utility in pharmacologic and diagnostic applications (86). Upon binding to BAFF, the extracellular domain of BAFF-R is processed by a metalloprotease (ADAM10) only in cells that also express TACI (87). This regulated processing is different from that of TACI and BCMA, receptors that undergo constitutive processing to release soluble fragments (88, 89). The BAFF trimer induces processing of BAFF-R by ADAM10, whereas TACI processing is unaffected by BAFF trimer stimulation (87). BAFF 60-mers are capable of stimulating processing of BAFF-R and TACI by both ADAM10 and ADAM17 (87). In the same study, the two metalloproteases, ADAM10 and ADAM17, demonstrated differential activity with respect to the activity state of B cells with increased ADAM10 activity on resting and TLR9-activated B cells, and ADAM17 processes BAFF-R on dark zone and germinal center B cells. Inhibition of ADAM10, responsible for processing of BAFF-R and TACI, was then shown to increase BAFF-dependent survival and secretion of IgM from B cells.

TACI is constitutively processed by ADAM10 on the surface of B cells to release the soluble extracellular domain of TACI capable of binding to BAFF and APRIL (88). Then γ-secretase, an intramembranous protease, cleaves the remaining membrane-proximal TACI fragment to prevent receptor-dependent activation of canonical NF-κB signaling (88). There is conflicting evidence supporting the capacity of the extracellular domain of TACI fused to an immunoglobulin Fc domain (TACI-Fc) to induce reverse signaling in macrophages through membrane bound BAFF and APRIL (90, 91). Studies that interrogate the role of soluble TACI must take into consideration differences in amino acid composition of endogenous sTACI compared to that of TACI-Fc due to demonstrated differences in BAFF/APRIL binding between sBCMA and BCMA-Fc (89). Subtle differences in the amino acid composition may have drastic effects on ligand binding capacity of the extracellular domain, as point mutations in TACI are capable of diminishing affinity for ligand, processing of TACI, and even processing of BAFF-R (87). Thus, the biological impact of soluble TACI remains incompletely understood.

BCMA is constitutively processed by γ-secretase, a process that acts to reduce surface BCMA and consequently regulate the number of plasma cells in the bone marrow, given the importance of this receptor for plasma cell survival (89). Although BCMA is able to bind BAFF and APRIL to induce canonical NF-κB signaling, soluble BCMA (sBCMA) is able to bind APRIL but does not block BAFF-mediated activation of NF-κB in HEK cells transfected with BCMA (89). The same study also found recombinant BCMA-Fc to bind BAFF and APRIL, leading to inhibition of BAFF and APRIL-mediated NF-κB signaling through BCMA. Quantification of serum BCMA revealed markedly reduced levels among patients with severe PAD, such as CVID and XLA (92). Evaluation of immunoglobulin deficiencies in CVID and other PADs often requires repeated vaccine challenges and discontinuation of immunoglobulin replacement therapy, which increase patient susceptibility to infection and may take several weeks (93–95). Methods of diagnosing PAD requiring immunoglobulin replacement that reduces diagnostic delay and does not require treatment discontinuation, such as is the case with sBCMA measurement, could significantly improve clinical care and quality of life in those with PAD.

## The Potential Contribution of BAFF to CVID ILD

CVID patients can have a significant increase in serum IgM corresponding to progression of ILD as determined by pulmonary function decline (96). This serum IgM increase is associated with hyperplasia of ectopic pulmonary B cells expressing IgM (28). B cell depletion with rituximab ameliorates CVID ILD, corresponding with improved pulmonary function and reduction of serum IgM, compared to those receiving supportive care (28). Moreover, the ILD recurrence that occurred in 1/3^rd^ of study subjects within 2 years of receiving rituximab was also associated with serum IgM elevation (28). Thus, the presence and reemergence of B cells, corresponding with rising levels of serum IgM, may be quite fundamental to CVID ILD pathogenesis.

CVID patients who experienced ILD progression after rituximab had significantly elevated levels of BAFF in blood and lung tissue compared to CVID patients with stable ILD, no ILD, and healthy controls (28). IFN-γ upregulates signal transducer and activator of transcription 1 (STAT1) expression to act as a potent stimulus of BAFF production (97). Numerous reports that have found elevation of IL-12, IFN-γ, and related T helper type 1 cytokines in CVID patients with inflammatory complications (28, 98–105). Furthermore, plasma IFN-γ levels and STAT1 expression were elevated in CVID patients with progressive ILD and correlated with BAFF expression, and CD14^+^ monocytes were identified as a prominent source of IFN-y-induced BAFF production and STAT1 expression in CVID patients with progressive ILD (28). Together, these results implicate an IFN-γ:STAT1:BAFF axis in pathogenesis of ILD in CVID. Efforts to unravel fundamental biology and clinical importance of this IFN-γ and BAFF relationship in CVID are underway.

Heterozygous mutations of TACI found in CVID appear to be key for the persistence of autoreactive B cells through interaction with toll-like receptor (TLR) 7 and TLR9 (106). Moreover, when BAFF is elevated in non-CVID patients it has been shown that autoantigen-engaged B cells demonstrate enhanced survival and migration to follicular zone and marginal zone niches where they would normally be excluded (107, 108). While the relationship between B cell autoreactivity and ILD is unclear in CVID, it is possible that enhanced BAFF-R signaling in the absence of counterbalancing signals from TACI promotes pathogenic pulmonary B cell hyperplasia. Indeed, 3 patients with TACI mutations in our study of CVID all had progressive ILD that recurred after rituximab (28). Thus, in addition to the greater prevalence of progressive ILD in CVID patients with TACI mutations there was apparently greater resistance to B cell depletive therapy, possibly due to elevated signaling through BAFF-R.

BAFF-R is the predominant BAFF receptor expressed by the IgD+ B cells that make up the ectopic pulmonary follicles observed in CVID ILD, while TACI is expressed in the extrafollicular areas of the lung harboring plasmablasts expressing IgM and the proliferation marker Ki67 (28). BAFF-R is the principal BAFF receptor on B cells in CVID patients with autoimmune and lymphoid hyperplasia due to the lack of marginal zone, memory, and plasma cells in these patients that would otherwise express TACI and/or BCMA (109, 110). Elevated levels of BAFF enhance BAFF-R-mediated activation of the non-canonical NF-κB pathway to upregulate Bcl-2 survival signals and impair B cell apoptosis (45, 48). The expanded subset of naïve B cells in CVID ILD were observed to induce expression of Bcl-2 and RelB to a level that is significantly greater in CVID patients with progressive ILD compared to healthy controls (28). Enhanced activity of BAFF-R signaling in response to elevated BAFF not only drives proliferation and resistance to apoptosis in naïve B cells, but may concurrently impair B cell maturation by drowning out BAFF-mediated maturation signals from TACI (45, 63). Excessive BAFF inhibits autophagy in B cells and reduces autophagosome marker LC3-II through mechanisms that depend on active Akt/mTOR signaling, suggesting that elevated BAFF can drive B cell survival through multiple mechanisms (111).

The extent of B cell contributions to pathogenesis of CVID ILD remains to be sufficiently defined. Like B cells, T cells are a prominent feature of CVID ILD pathology, and treatment with azathioprine or mycophenolate mofetil in combination with rituximab improved clinical chest radiography scores and components of pulmonary function testing in patients with CVID ILD (15). A considerable portion of patients in this study relapsed after receiving this immunosuppressive combination in association with elevated B cells and activated CD4^+^ T cells. Variations in the extent of immune cell compartment imbalance in CVID may enhance the progression of ILD due to mechanisms that remain unclear. T cells from CVID patients demonstrate increased frequencies of activated, memory, and effector populations with a lack of naïve and regulatory T cell subsets (112). The enhanced state of T cell activation and effector function in CVID may further contribute to the B cell hyperplasia observed in CVID ILD due to a lack of T cell-mediated regulation of B cell activity in addition to upregulation of non-canonical NF-κB signaling in B cells as a result of more widespread stimulation of CD40 through CD40L expressed on activated T cells (113, 114). The notable variability of clinical manifestations and aberrant immune cell compartments in CVID suggests that multiple aspects of immune system dysregulation may contribute to CVID ILD. Furthermore, efficacy of therapeutic depletion of B cells may stem from indirect effects upon leukocytes, such as T cells, that closely interact with B cells in CVID ILD.

Studies of lung disease with pathologic similarities to that observed in CVID ILD may also prove to be informative. For example, lymphocytic interstitial pneumonia makes up 15% of interstitial lung disease affecting Sjogren’s syndrome patients (115). Similar to CVID, B cells appear to play a central role in the development of ILD in Sjogren’s syndrome. Specifically, elevated levels of BAFF can be found in the serum, saliva, and salivary glands of Sjogren’s syndrome patients in comparison to healthy controls (116–118). BAFF levels in these patients are also positively associated with the presence of autoantibodies, including anti-SSA and anti-SSB (119). Also, like we found in CVID ILD, elevated levels of BAFF seen in Sjogren’s is associated with heightened interferon signaling through the JAK/STAT pathway in monocytes (28, 120). Elevated levels of BAFF in Sjogren’s syndrome ultimately enables prolonged survival of B cells, which have been shown to aggregate into inducible bronchus-associated lymphoid tissue structures with pulmonary B cell follicles as in CVID ILD (121). A double-blind, randomized, placebo-controlled, multi-center, multi-national clinical trial (NCT02631538) that investigated the effects of rituximab and belimumab administration in 86 pSS patients was recently completed in June 2020. This trial contained four groups, including a placebo group, a group that received only belimumab, a group that received only rituximab, and a group that received both belimumab and rituximab. Results from this study have not been published yet, but they will put the implication of BAFF and aberrant B cell survival and signaling found in Sjogren’s syndrome patients to the test.

Another chronic lung disease where there is increasing evidence for a role of B cells and BAFF is chronic obstructive pulmonary disease (COPD). Although COPD is commonly associated with smoking, anywhere between 25 and 45% of COPD patients have never smoked, suggesting that other factors contribute to the pathogenesis of this lung disease (122). The implication of the adaptive immune system in the development and progression of COPD becomes evident when considering the fact that there is a significantly greater number of B cells and CD4+ and CD8+ T cells in the airways and parenchyma of the lungs of COPD patients (123, 124). These excess B and T cells arise from induced bronchus-associated lymphoid tissue (iBALT) and form pulmonary follicles containing germinal center B cells and follicular T cells (123). Moreover, significantly more lymphoid follicles were found in the lungs of those who were diagnosed with COPD in comparison to smokers without COPD (124). Also, when categorizing COPD patients on the Global Initiative for Chronic Obstructive Lung Disease (GOLD) scale, a significant increase in the number and size of lymphoid follicles was seen in later-stage COPD patients in comparison to those in the earlier stages (124). The same study also performed immunofluorescence on lung samples and found the size of the lymphoid follicles identified in each of the aforementioned groups to be directly correlated to the percentage of BAFF-positive B cells, which co-localized with BAFF-R (124, 125). BAFF expression was also found to be elevated in the blood of COPD patients in comparison to non-smoking and smoking control subjects (124). Healthy, smoking controls and the early-stage COPD subjects, on the other hand, had a higher proportion of caspase-3-positive B cells, indicating apoptosis, in their pulmonary follicles in comparison to later-stage COPD subjects. These findings implicate dysregulation of the BAFF : BAFF-R axis in the progression of COPD, with the anti-apoptotic signals of BAFF-R promoting the B cell follicles that are a major component of pulmonary pathology, similar to what was found in CVID ILD.

## Conclusion

There is increasing evidence that dysregulated B cell responses, such as those exacerbated by BAFF, promote the progression of ILD in CVID. This is supported by the adoption of B cell depletive therapy, either alone or in combination with other immunosuppression, as a fundamental component of CVID ILD treatment. Continued suppression of B cell activation through administration of immunosuppressive antimetabolite agents such as azathioprine or mycophenate, or potentially through inhibition of BAFF may help maintain CVID ILD in remission. B cell hyperplasia is a defining aspect of CVID ILD and is perpetuated *via* survival signals mediated by BAFF through BAFF-R. In addition to B cells, CVID ILD consists of prominent T cell infiltration which appears to also improve with B cell depletive therapy (23, 28). The link between B cells and T cells in the CVID lungs remains undefined, and whether depletion of B cells removes a vital antigen-presenting cell, lymphoid structure, source of chemokines, and/or another component required for T cell recruitment and persistence in the lungs is unknown. Further research is necessary to prove whether B cells fundamentally contribute to pathogenesis of CVID ILD, define the best way to achieve safe long-lasting suppression of dysregulated B cell responses, and accurately identify the individual CVID patients who would most benefit from B cell-targeted therapy. Moreover, we must elucidate mechanisms by which the IFN-γ/STAT1/BAFF axis is elevated in CVID and other disorders. Further efforts to unravel the mechanisms by which BAFF and B cells become dysregulated in CVID offer potential to address these knowledge gaps in CVID and other forms of autoimmune and inflammatory disease.

## Author Contributions

EM, MA, KB, and KH drafted the manuscript. PM provided guidance and revisions. All authors contributed to the article and approved the submitted version.

## Funding

This work was supported by National Institutes of Health grants AI137183 and AI151486, a faculty development grant from the American Association of Allergy, Asthma and Immunology Foundation, and a Boston University Career Investment Award.

## Conflict of Interest

The authors declare that the research was conducted in the absence of any commercial or financial relationships that could be construed as a potential conflict of interest.

## Abbreviations

APRIL, a proliferation-inducing ligand; BAFF, B cell activating factor; BAFF-R, BAFF receptor; BCMA, B cell maturation antigen; COPD, chronic obstructive pulmonary disease; CSR, class-switch recombination; CTLA-4, cytotoxic T-lymphocyte-associated protein 4; CVID, common variable immunodeficiency; GLILD, granulomatous lymphocytic interstitial lung disease; GOLD, Global Initiative for Chronic Obstructive Lung Disease; IKKα, IκB kinase; iBALT, induced bronchus-associated lymphoid tissue; ILD, interstitial lung disease; LRBA, lipopolysaccharide (LPS)-responsive and beige-like anchor protein; NIK, NF-κB-inducing kinase; PAD, primary antibody deficiency; STAT1, signal transducer and activator of transcription 1; TACI, transmembrane activator and CAML interactor; TI, T cell-independent; TRAF3, TNF receptor-associated factor 3.
